# Grazing alters species relative abundance by affecting plant functional traits in a Tibetan subalpine meadow

**DOI:** 10.1002/ece3.7891

**Published:** 2021-07-27

**Authors:** Qifang He, Kai Jiang, Weicheng Hou, Yang Zhao, Xinhang Sun, Lu Wang, Yike Zou, Zhenhao Zhu, Hui Zhang

**Affiliations:** ^1^ Key Laboratory of Genetics and Germplasm Innovation of Tropical Special Forest Trees and Ornamental Plants (Hainan University) School of Forestry Ministry of Education Hainan University Haikou China; ^2^ School of Forestry, Wuzhishan National Long Term Forest Ecosystem Monitoring Research Station Hainan University Haikou China

**Keywords:** aboveground and belowground traits, grazing, resource‐based trade‐offs, restoration, subalpine meadows, trait–abundance relationships

## Abstract

Domestic livestock grazing has caused dramatic changes in plant community composition across the globe. However, the response of plant species abundance in communities subject to grazing has not often been investigated through a functional lens, especially for belowground traits. Grazing directly impacts aboveground plant tissues, but the relationships between above‐ and belowground traits, and their influence on species abundance are also not well known. We collected plant trait and species relative abundance data in the grazed and nongrazed meadow plant communities in a species‐rich subalpine ecosystem of the Qinghai–Tibet Plateau. We measured three aboveground traits (leaf photosynthesis rate, specific leaf area, and maximum height) and five belowground traits (root average diameter, root biomass, specific root length, root tissue density, and specific root area). We tested for shifts in the relationship between species relative abundance and among all measured traits under grazing compared with the nongrazed meadow. We also compared the power of above‐ and belowground traits to predict species relative abundance. We observed a significant shift from a resource conservation strategy to a resource acquisition strategy. Moreover, this resource conservation versus resource acquisition trade‐off can also determine species relative abundance in the grazed and nongrazed plant communities. Specifically, abundant species in the nongrazed meadow had aboveground and belowground traits that are associated with high resource conservation, whereas aboveground and belowground traits that are correlated with high resource acquisition determined species relative abundance in the grazed meadow. However, belowground traits were found to explain more variances in species relative abundance than aboveground traits in the nongrazed meadow, while aboveground and belowground traits had comparable predictive power in the grazed meadow. We show that species relative abundance in both the grazed and the nongrazed meadows can be predicted by both aboveground traits and belowground traits associated with a resource acquisition versus conservation trade‐off. More importantly, we show that belowground traits have higher predictive power of species relative abundance than aboveground traits in the nongrazed meadow, whereas in the grazed meadows, above‐ and belowground traits had comparable high predictive power.

## Graphical abstract

Species abundance with and without intensive grazing can be predicted by both aboveground and belowground traits that are associated with a resource acquisition vs. resource conservation trade‐off. Intensive grazing can alter the predictive abilities of aboveground and belowground traits for species abundance.

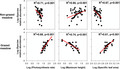

## 1 INTRODUCTION

Understanding the impact of disturbance on community assembly remains a key challenge in ecology and conservation science (Mouillot et al., 2013). Grazing by domestic livestock is one of the most globally widespread land uses, which has led to dramatic changes in plant community composition and species diversity (Chapin et al., 2000; Li et al., 2017). Thus, there is an urgent need to better understand how grazing alters plant community assembly—if we are to restore plant diversity in the grazed meadows (Wang et al., 2018).

Resources acquisition versus resource conservation trade‐off are the key to lead to varied plant community compositions, and plant species with high resource acquisition generally increase in relative abundance after grazing (Augustine & McNaughton, 1998; Díaz et al., 2001; Evju et al., 2009). In contrast, plant species with high resource conservation tend to dominate the nongrazed meadows (Zhang et al., 2015; Zhang, Chen, et al., 2018; Zhang, John, et al., 2018). Indeed, a meta‐analysis has found that at the global scale, annual species are the dominating species in the grazed meadows; in contrast, perennial species dominate the nongrazed meadows (Díaz et al., 2007). Usually, annual species tend to have high resource acquisition, whereas perennial species tend to promote high resource conservation strategy (Roumet et al., 2006, 2016; Zhang, Chen, et al., 2018; Zhang, John, et al., 2018). The shifts from resource conservation in the nongrazed meadow to resource acquisition in the grazed meadow can result in responding to varied functional traits (presumed adaptive morphological and physiological traits; Strauss & Agrawal, 1999). For example, higher resource acquisition rates in the face of high grazing pressure tend to make plant to have shorter plant maximum height and higher specific leaf area (Díaz et al., 2001; Li et al., 2017), whereas high resource conservation rates in the nongrazed condition tend to make plants to have lower specific leaf area and taller stature to adapt to resource limitation (Díaz et al., 2007). Thus, functional traits can have high power in predicting grazing‐induced variations in species abundances (Niu et al., 2010). However, other studies analyzing the response of Australian dry shrub and wood lands found that functional traits show very low power to predict variation in plant species abundance in response to grazing (Vesk et al., 2004). Moreover, research in West African savannas, semiarid Australian shrublands, subhumid grasslands, and subalpine and alpine meadows (Cingolani et al., 2005; de Bello et al., 2005; Díaz et al., 2007; Kahmen et al., 2002; Klimesova et al., 2008; Kühner & Kleyer, 2008; Li et al., 2013; Meers et al., 2008; Niu et al., 2010; Wang et al., 2018; Yang et al., 2015) has failed to reach a consensus that functional traits are indeed good indicators of grazing‐induced differences in species abundance.

Two main problems persist when investigating trait–abundance relationships: Comparisons between the grazed and the nongrazed communities that focus mainly on aboveground traits (Niu et al., 2010) and belowground traits remain overlooked in grazing‐related studies and global databases (Bergmann et al., 2017). Belowground traits have been found to be highly correlated with a resource acquisition versus. resource conservation trade‐off (de la Riva et al., 2018; Prieto et al., 2015) and thus may also play a key role in mediating grazing‐induced alterations in community composition (Klumpp et al., 2009; McInenly et al., 2010). However, belowground traits do not necessarily fall in the same strategy gradients as aboveground traits (Bergmann et al., 2017, 2020). Moreover, there is little study reporting the strong belowground trait–abundance relationships in both the grazed and the nongrazed meadows. Thus, quantifying relationships between species relative abundance and both of aboveground and belowground traits in the grazed and nongrazed communities may help to understand how grazing modifies community assembly processes.

Compared with aboveground traits, belowground traits are much harder to measure, but belowground traits can better capture plant resource conservation than aboveground traits, as they can better reflect a plant's ability to acquire limiting nutrients (e.g., nitrogen; Bardgett et al., 2014). However, it has been found that both aboveground and belowground traits can shift from resource conservation in the nongrazed meadow to resource acquisition in the grazed meadow (Roumet et al., 2016). Moreover, strong correlations between aboveground and belowground traits have been reported (de la Riva et al., 2018). Aboveground traits are thus assumed a good proxy for belowground traits (Klumpp et al., 2009). If this is true, merely using aboveground traits is useful enough to understand how grazing‐induced shifts in a resource acquisition versus resource conservation trade‐off affect community assembly. If this is not true, both belowground and aboveground traits should be measured; otherwise, using aboveground trait only cannot be good enough to revel how grazing‐induced shifts in resource acquisition and resource conservation strategies influence community assembly. Quantifying the relative contribution of aboveground and belowground traits to species relative abundance can help verify this, but nearly no study has investigated the relative importance of aboveground traits and belowground traits in species relative abundances in the nongrazed and grazed meadows.

Here, we compared plant functional traits in a nongrazed meadow and a grazed meadow in the Qinghai–Tibet Plateau—both of which have 30 years of well‐documented grazing history. We collected an extensive dataset consisting of: (a) three plant aboveground traits (maximum photosynthesis rate, specific leaf area, and maximum height); (b) five plant belowground traits (root diameter, root biomass, specific root length, root tissue density, and specific root area); and (c) plant species relative abundance. The selection of these traits was based on their strong associations with plant resource acquisition and resource conservation strategies (de la Riva et al., 2018). For example, shorter plant maximum height and higher specific leaf area are associated with high resource acquisition despite high grazing pressure (Díaz et al., 2001; Li et al., 2017). In contrast, in the nongrazed condition, lower specific leaf area and higher plant maximum height may be a strategy for possessing high resource conservation to adapt to resource limitation (Westoby et al., 1999). The grazed communities generally had higher light availability (Rook et al., 2004) than the nongrazed communities and thus may have different photosynthesis rate–abundance relationships. Moreover, higher photosynthesis rate is indicative of higher growth rates (Kirschbaum, 2011; Zhang, John, et al., 2018) and thus may reflect plant resource acquisition. Belowground traits often exhibit a resource acquisition versus resource conservation trade‐off, with resource acquisition warranting high specific root length and specific root area, but low root tissue density, root biomass, and root density; and resource conservation resulting in high root tissue density, root biomass, and root density, but low specific root length and specific root length (Feng et al., 2018; Klumpp et al., 2009; McInenly et al., 2010; Roumet et al., 2016). However, it remains unknown whether these aboveground and belowground traits can determine species relative abundance in the nongrazed and grazed meadows.

Employing this extensive dataset, our goal was to evaluate the effect of excluding grazing from an ecosystem with a long history of both managed grazing and utilization by native herbivores. Specifically, we aim to quantify the following: (a) whether grazing and grazing removal alter plant aboveground and belowground traits so that they can shift from resource conservation to resource acquisition in Qinghai–Tibet Plateau; (b) whether the resource acquisition versus resource conservation trade‐off reflected in both aboveground and belowground traits can determine species relative abundance in the nongrazed and grazed meadows; and (c) whether aboveground and belowground traits have the comparable predictive power of above‐ and belowground traits on species relative abundance in the grazed and nongrazed meadows.

## 2 METHODS

### 2.1 Study site

Field sampling was conducted in two species‐rich subalpine meadows located in the eastern section of the Qinghai–Tibet Plateau, Luqu, China (34°5′N, 102°10′E, Figure S1a). Mean annual precipitation in the region is ~530 mm, 70% of which occurs from June to August. The mean annual temperature is 2.4°C. Vegetation at the study sites is dominated by *Elymus nutans*, *Kobresia humilis*, and *Thermopsis lanceolata*, and soils are classified as alpine meadow soils (Zhang, John, et al., 2018). The focal meadows are in a large area of 4,000 ha.

### 2.2 Sampling

We sampled one nongrazed meadow (control) and one grazed meadow. The land‐use histories over the last 30 years for the grazed and nongrazed meadows were obtained by interviewing local farmers. Since year 2000, the Chinese government has established the fence to protect some areas of this grazed meadow, thereby resulting in a nongrazed meadow. Thus, the nongrazed meadow (also fenced meadow) had not been grazed for 18 years prior to the study. Yak is the only disturbance, and grazing by small mammals has not been reported or observed in either the grazed or the nongrazed meadow.

We placed 30 0.25‐m^2^ quadrats in each of the grazed and nongrazed plots (Figure S1b), in August (peak growing season) 2018. Quadrats were regularly spaced at 20‐m intervals along 6 parallel transects. The grazed and nongrazed plots were about 500 m distant. Species relative abundance was measured as number of individuals and biomass (Morlon et al., 2009), because many meadow plant species are clonal and aboveground biomass may better reflect species relative abundance than considering only the number of individuals. To quantify aboveground biomass for each species, we harvested all aboveground parts for each species and oven‐dried them at 80°C for 2 days before weighing. Species relative abundance was then calculated as the ratio of aboveground biomass of each species to the total aboveground biomass of all species in all 30 0.25‐m^2^ quadrats in the nongrazed and grazed meadows, respectively.

### 2.3 Functional trait measurements

Fifteen mature individuals were randomly selected for each species recorded in the 30 quadrats of 0.25 m^2^ in the grazed and nongrazed meadows, respectively, for measurements of three aboveground traits (maximum photosynthesis rate [A; µmol m^−2^ s^−1^], specific leaf area [cm^2^/g], and plant maximum height [cm]) and five belowground traits (root average diameter [mm], root biomass [g], specific root length [cm/g], root tissue density [g/cm^−3^], and specific root area [cm^2^/g]) (Table S2). Measurements were done as described in Zhang, John, et al. (2018) and Bergmann et al. (2017), and further details are given in the Supplementary Material (Text S1). Specifically, the 30 quadrats were used to generate a species list, and then, each species was assigned a mean value for each trait based on the measurements of 15 individuals per species. This process was repeated for both the grazed and the nongrazed treatments. We had 42 species that were present in the nongrazed and grazed meadows, and we measured their traits in all the meadows. This way, we hoped to ensure that intraspecific variation was appropriately incorporated in our analyses.

### 2.4 Statistical analysis

We firstly used a Pearson correlation to examine the bivariate relationships among all aboveground and belowground plant traits for all species found in the nongrazed and grazed meadows. We secondly run a principal component analysis (PCA) to evaluate whether species trait values varied between the grazed and the nongrazed meadows can be significantly differentiated by these eight aboveground and belowground traits. We thirdly tested whether species relative abundance for all species in the nongrazed and grazed meadows can be well predicted by PCA axis (PC1 and PC2) using linear regression. We also examined whether species relative abundance of each plant species can be well predicted by its responding aboveground and belowground traits in the nongrazed and grazed meadows individually using linear regression. Finally, we used a variance partitioning analysis to quantify the relative contribution of aboveground and belowground traits to species relative abundance for all species in the grazed and nongrazed meadows, thereby revealing whether aboveground traits can be a good proxy for belowground traits in predicting species relative abundance for all species in the nongrazed and grazed meadows, respectively. Specifically, species relative abundance with traits as explanatory variables can be divided into four complementary components: (a) “purely aboveground traits,” variance explained by aboveground traits alone; (b) “shared aboveground and belowground traits,” variance explained by both aboveground traits and belowground traits; (c) “purely belowground traits,” variance explained by belowground traits alone; and (d) “unexplained residual variation” (Legendre et al., 2009; Zhang, Chen, et al., 2018). For both the nongrazed and the grazed meadows, variance partitioning was done using the function “varpart” in the “*vegan*” R package (Oksanen et al., 2016). The percentage of variation is achieved by adjusted R‐square from variance partitioning (Legendre et al., 2009).

Our analyses were parametric and assumed normally distributed data. Across all sites, both species relative abundance and trait values were strongly right‐skewed, so we log‐transformed both species relative abundance and trait data, which resulted in near‐normal distributions for both.

## 3 RESULTS

We sampled 63 and 46 native species in the nongrazed and grazed meadow, respectively (Table S1). Species richness was higher in the nongrazed meadow than in the grazed meadow, and 42 of the 46 species in the grazed meadow were also present in the nongrazed meadow (Table S1). However, species composition differed: Native perennial species dominated the nongrazed meadow, while native annual species were more prominent in the grazed meadow (Figure S2).

**FIGURE 1:**
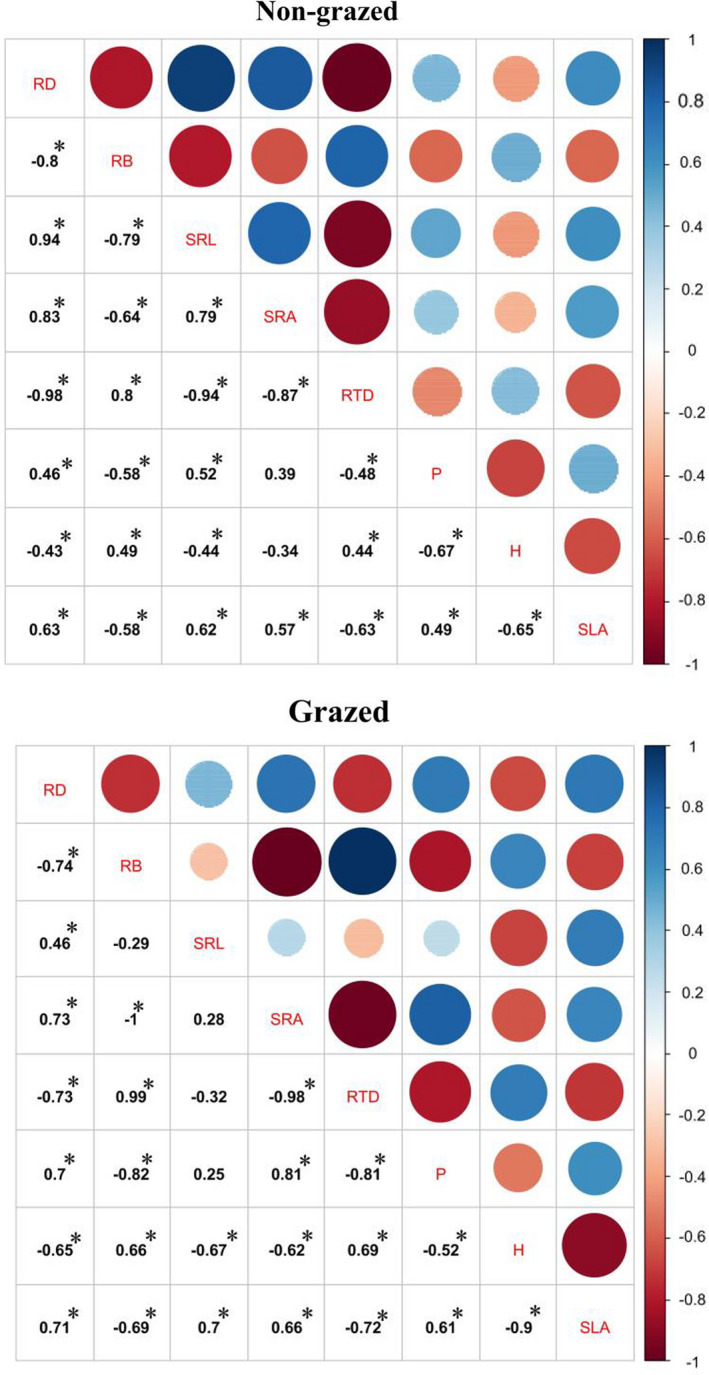
Interrelationships among aboveground traits (maximum height, photosynthesis rate, and specific leaf area) and belowground traits (root density, root biomass, specific root length, specific root area, and root tissue density) in the nongrazed and grazed meadows. * indicates *p* < .05 based on correlation analysis

We observed strong correlations among the five root traits, as well as between the three aboveground traits, for all species in the nongrazed and grazed meadows (Figure 1). For example, root density, maximum height, root biomass, and root tissue density are all significantly positively correlated (Figure 1). Similarly, specific root length, photosynthesis rate, specific root area, and specific leaf area are also all significantly positively correlated (Figure 1). However, RD, H, RB, and RTD are significantly negatively associated with specific root length, photosynthesis rate, specific root area, and specific leaf area (Figure 1).

**FIGURE 2:**
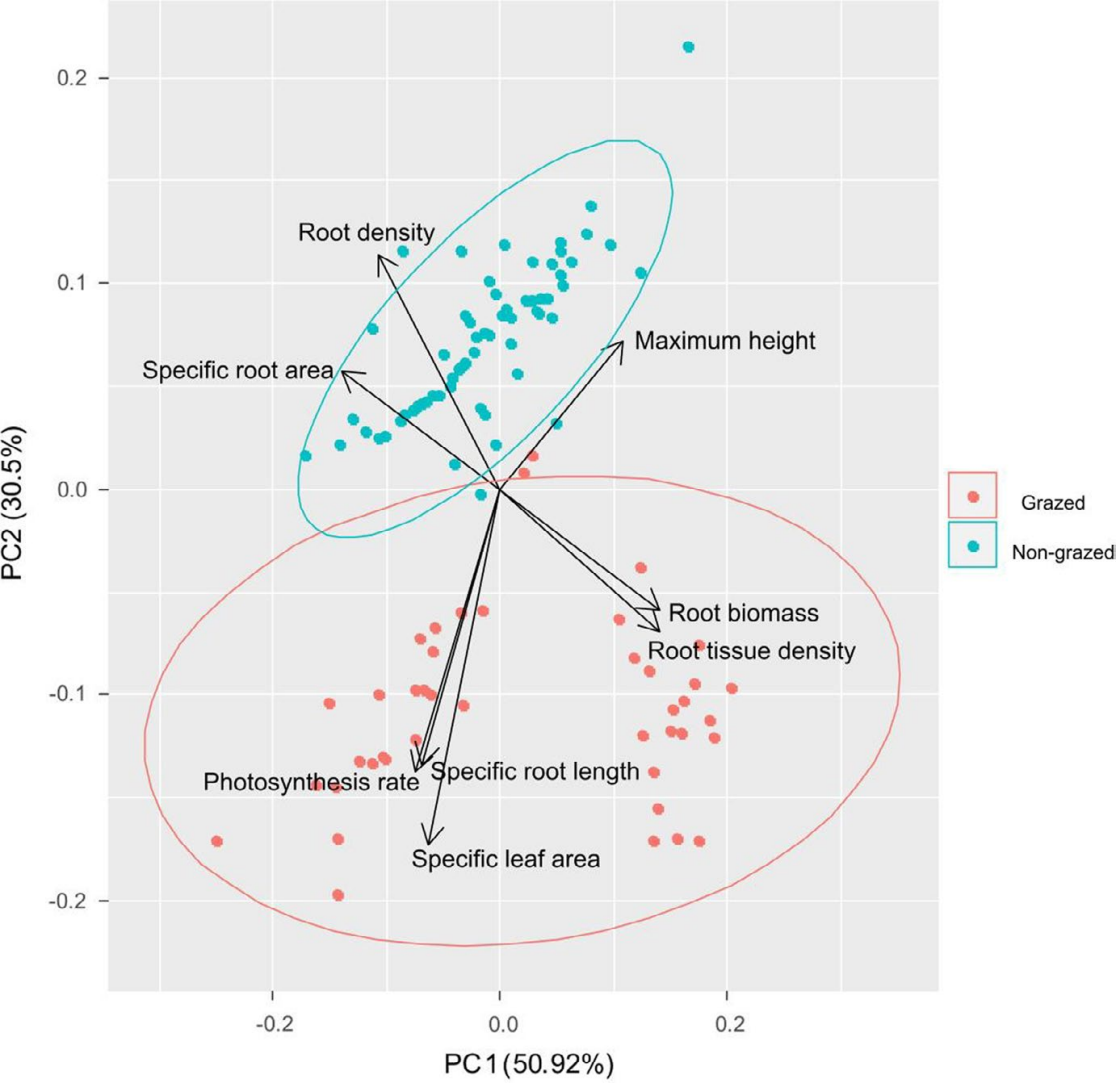
Principal component analysis of belowground (root density, root biomass, specific root length, specific root area, and root tissue density) and aboveground traits (maximum height, photosynthesis rate, and specific leaf area) in the nongrazed and grazed meadows

Results of PCA revealed that the “nongrazed and grazed species” were significantly separated by PC2 (Table S3 and Figure 2). PC2 was also good predictor of species relative abundance in both the grazed and the nongrazed meadows (Figure S3).

**FIGURE 3:**
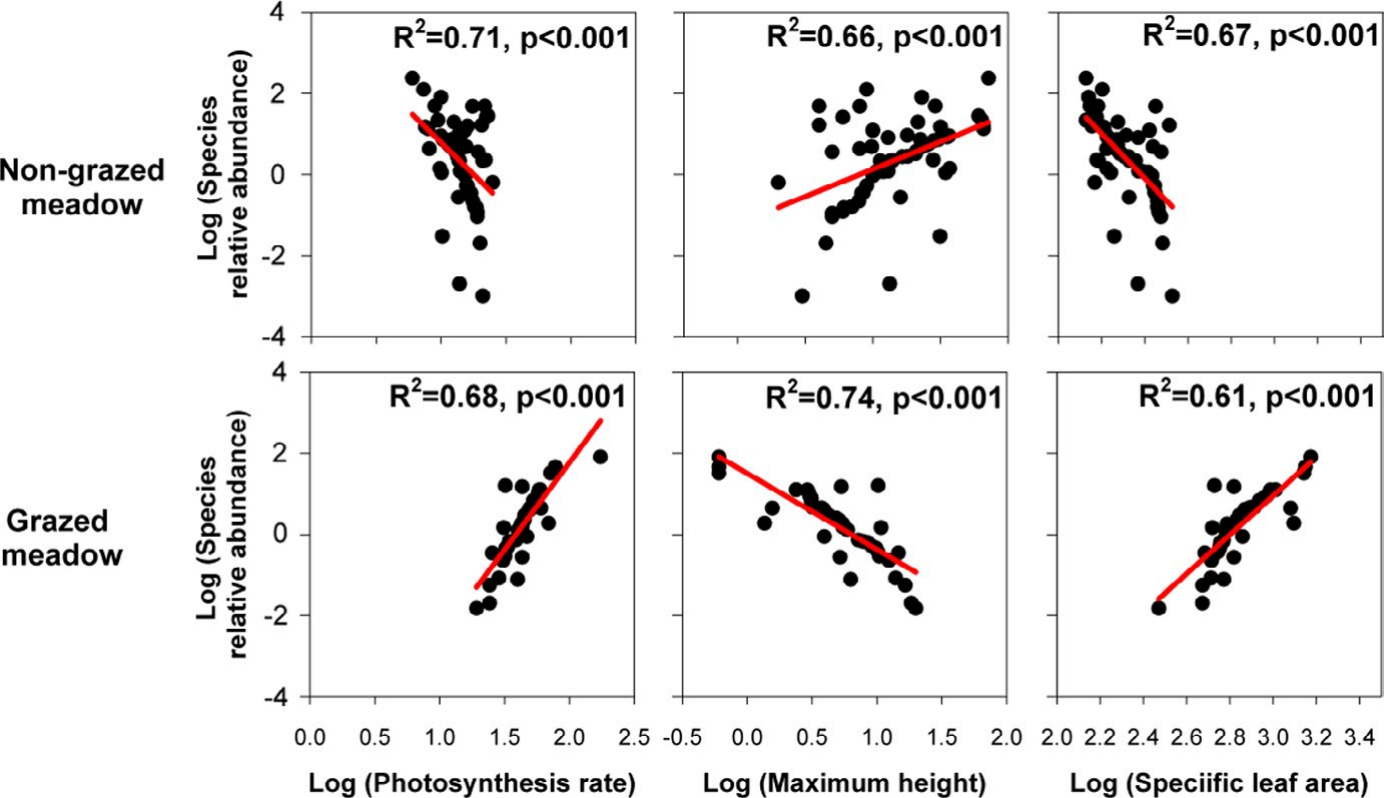
Relationships between species relative relative abundance (%) and aboveground traits: photosynthetic rate (µmol m^−2^ s^−1^), plant maximum height (cm), and specific leaf area (cm^2^/g) in the nongrazed (above) and grazed (below) meadows. Each point represents the mean value of a single species. Fitted red lines are generated from linear regression with corresponding significance (P)

**FIGURE 4:**
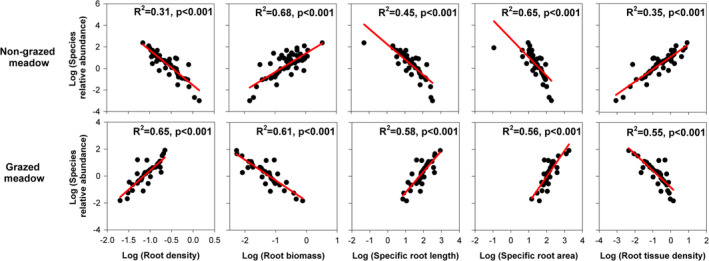
Relationships between species relative abundance and five measured belowground traits: root average diameter (mm), root biomass (g), specific root length (cm/g), root tissue density (g/cm^−3^), and specific root area (cm^2^/g) in the nongrazed (above) and grazed (below) meadows. Each point represents the mean value of a single species. Fitted red lines are generated from linear regression with corresponding significance (P)

All aboveground and belowground traits were good predictors of species relative abundance in both the heavily grazed and the nongrazed meadow communities (*p* < .001; Figures 3 and 4). The relationships between species relative abundance and plant traits were often in opposite directions in the nongrazed and grazed meadows. Abundant species in the nongrazed meadow exhibited relatively high maximum height, root biomass, and root tissue density, but low photosynthesis rate, specific leaf area, specific root length, and specific root area—the converse was true for nonabundant species (*p* < .001; Figures 3 and 4). In contrast, in the grazed meadow, abundant species had high photosynthesis rate, specific leaf area, specific root length, and specific root area, but low maximum height, root biomass, and root tissue density, and the opposite patterns were evident for nonabundant species (*p* < .001; Figures 3 and 4).

**FIGURE 5:**
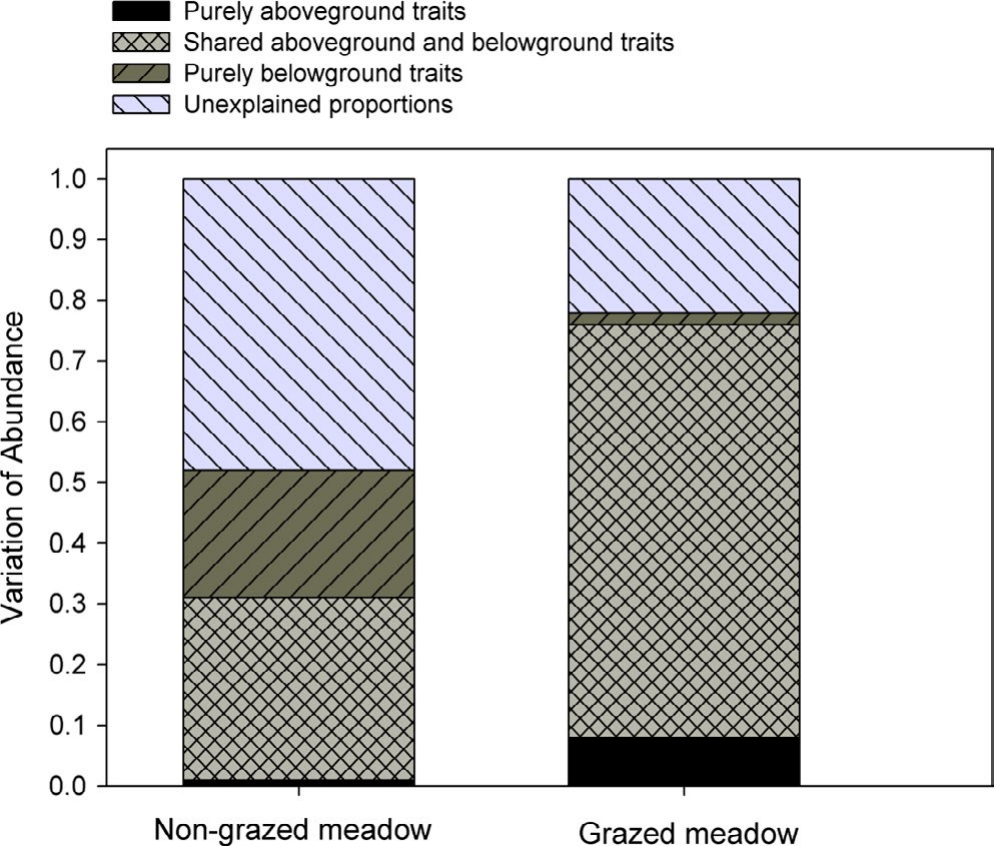
The percentage of variation in species relative abundance explained by four predictor types: (a) “purely aboveground traits”; (b) “shared aboveground and belowground traits,” variance explained by both above‐ and belowground traits; (c) “purely belowground traits”; and (d) “unexplained variables” in the nongrazed and grazed meadow, respectively. The percentage of variation is achieved by adjusted *R*‐square from variance partitioning

Variance partitioning analyses showed that in the nongrazed meadows, belowground traits explained a greater proportion of the variance (51%) than aboveground traits (31%) in predicting species relative abundance (Figure 5). However, in the grazed meadows, above‐ and belowground traits had comparable high predictive power of species relative abundance (76% and 70% of the total variance, respectively; Figure 5).

## 4 DISCUSSION

This study provides empirical evidence that grazing and grazing removal alter plant aboveground and belowground traits so that they can shift from resource conservation in the nongrazed meadow to resource acquisition in the grazed meadow. Meanwhile, the resource acquisition versus resource conservation trade‐off reflected in both aboveground and belowground traits can also determine species relative abundance in the nongrazed and grazed meadows. However, aboveground and belowground traits have different relative power in predicting species relative abundance in the grazed and nongrazed meadow communities.

It has been widely found that the shift from resource conservation to resource acquisition can lead to significant relationships among aboveground and belowground traits at both the nongrazed and the grazed meadows (de la Riva et al., 2018; Prieto et al., 2015; Wright et al., 2004). Here, we found significant correlations among all aboveground and belowground traits we measured in both the nongrazed and the grazed meadows. This indicated that grazing and grazing removal altered plant aboveground and belowground traits so that they could shift from resource conservation to resource acquisition in the Qinghai–Tibet Plateau.

The principal component analysis revealed that the nongrazed and grazed species were significantly separated by PC2, whose lower and higher values are highly associated with a resource acquisition versus resource conservation trade‐off. Thus, the shift from resource conservation to resource acquisition led to significant different trait values between the nongrazed and the grazed meadows. We also found PC2 was significantly correlated with species relative abundances in both the nongrazed and the grazed meadows, indicating this shift from resource conservation to resource acquisition can also determine species relative abundance in both the nongrazed and the grazed meadows. As a result, the variations in aboveground and belowground traits that reflect a shift from resource conservation in the nongrazed meadow to resource acquisition in the grazed meadow may determine variations in species relative abundance in the nongrazed and grazed meadows. Indeed, we found annuals that had high resource acquisition‐dominated grazed meadow, and perennial species that possessed high resource conservation–dominated nongrazed meadows. We also found significant but differing trait–abundance relationships in the nongrazed and grazed meadows for both aboveground and belowground traits. Thus, the resource conservation versus resource acquisition trade‐off at both aboveground and belowground levels determined species relative abundance in both the nongrazed and the grazed meadows.

Given the strong correlations between aboveground and belowground traits and significant trait–abundance relationships for both aboveground and belowground traits, aboveground traits seem to be a good proxy of belowground traits in predicting species relative abundance in the nongrazed and grazed meadows. This is evident in the grazed meadows, as in the grazed meadows, aboveground and belowground traits explained comparable fractions of the variance (70% and 76%, respectively) in species relative abundance. Moreover, variance explained by both above‐ and belowground traits is very high (68%), which might be attributed to the strong correlations between aboveground and belowground traits. These results indicate that grazing may select for species with high resource acquisition strategies, possibly with a very narrow range of trait values, resulting in above‐ and belowground traits with comparable predictive power for species relative abundance. Compared with belowground traits, aboveground traits are relatively easier to measure and have been much more widely studied across global communities (Wright et al., 2004). Thus, we can only measure aboveground traits to quantify trait–abundance relationships in the grazed meadow in the future in the Qinghai–Tibet Plateau.

However, aboveground traits cannot be a good proxy for belowground traits in the nongrazed meadow. That is because in the nongrazed meadow, belowground traits explain a greater proportion of the variance (51%) than the aboveground traits (32%) in predicting species relative abundance. One possible reason is that resource competition may make resource conservation strategy prevail in the nongrazed meadows, which in turn may allow for a wider range of trait combinations, thereby leading to above‐ and belowground traits with different predictive power for species relative abundance. Indeed, our previous works have reported that competition in nitrogen uptakes was a major influence on community assembly in the nongrazed meadows in this subalpine ecosystem (Zhang et al., 2015; Zhang, Chen, et al., 2018). Perennials generally have larger and more established root systems than annuals (Roumet et al., 2006). Thus, compared with aboveground traits, plant's ability to acquire limiting nutrients can be better reflected by belowground traits (e.g., nitrogen; Bardgett et al., 2014). It is therefore not surprising that we found that belowground traits were stronger predictors of species relative abundance than aboveground traits in the nongrazed meadow. These findings imply that belowground traits may be more critical than aboveground traits for quantifying the role of the species niche on community assembly and maintenance of diversity in the nongrazed meadows. This indicates merely using aboveground traits only cannot reveal the strong effects of resource conservation on community assembly in the nongrazed meadows. Thus, both aboveground and belowground traits should be measured in the nongrazed meadows.

Despite these compelling results, our data had inherent limitation; namely, although we measured three aboveground and five belowground traits that capture grazing‐induced resource acquisition and resource conservation in the nongrazed meadows, foliar nutrient contents that may explain the unaccounted variances (22% and 48%, respectively) in species relative abundance should have been measured and tested. Many studies on the Tibetan Plateau involving fertilization treatments on the grazed and nongrazed meadow communities have established soil‐available nitrogen and phosphorus as critical limiting nutrients with substantial impacts on community structure (Niu et al., 2016; Yang et al., 2015; Zhang, Gilbert, Wang, et al., 2013; Zhang, Gilbert, Zhang, et al., 2013). There is no doubt that foliar nutrient contents are important and influence species relative abundance in these environments, which merit further investigation. Moreover, data on trait and species relative abundance and abiotic conditions (i.e., soil moisture and nutrient) at the nongrazed and grazed meadows should be collected over several time steps in the future, thereby providing insight as to whether these two sites are actually diverging in terms of species composition.

## 5 CONCLUSION

We provide novel insights on different contributions of above‐ and belowground traits to plant species relative abundance in the nongrazed and grazed meadows. Grazing disturbance is expected to affect mostly aboveground plant parts, but by imposing strong selection on the plant life histories that can survive under and sustained grazing, a wide range of aboveground and belowground plant traits are also selected. Moreover, such disturbance may select for a narrow range of trait values, whereas resource competition and persistence may allow for a wider range of trait combinations. However, future inter‐ and intraspecific trait variations should be quantified to confirm this. These findings need to be incorporated in the management of meadow plant communities under different kinds of land use. Our findings also provide clues about plant life histories suited for restoration of the grazed meadows for which natural recovery may be very slow or even stopped. For instance, active seeding of perennial species may lead to strong competition between perennial and annual species in the heavily grazed meadows, which may in turn quickly restore natural conditions.

## CONFLICT OF INTEREST

The authors declare no conflict of interest.

## AUTHOR CONTRIBUTIONS

**Qifang He:** Data curation (equal); Investigation (equal); Methodology (equal); Writing‐original draft (equal); Writing‐review & editing (equal). **Kai Jiang:** Data curation (equal); Formal analysis (equal); Methodology (equal); Writing‐review & editing (equal). **Weichen Hou:** Conceptualization (equal); Data curation (equal); Formal analysis (equal); Investigation (equal); Methodology (equal); Resources (equal); Validation (equal). **Yang Zhao:** Investigation (equal); Methodology (equal). **Xinhang Sun:** Conceptualization (equal); Data curation (equal); Formal analysis (equal); Investigation (equal); Methodology (equal); Validation (equal); Writing‐original draft (equal). **Lu Wang:** Investigation (equal); Methodology (equal). **Yike Zou:** Investigation (equal); Methodology (equal). **Zhenhao Zhu:** Investigation (supporting). **Hui Zhang:** Data curation (equal); Formal analysis (equal); Funding acquisition (equal); Investigation (equal); Methodology (equal); Project administration (equal); Supervision (equal); Validation (equal); Writing‐original draft (equal); Writing‐review & editing (equal).

## Supporting information

Appendix S1

## Data Availability

The data used in this manuscript have been provided in the Appendix S1.
