# Supplementary material for: Grazing alters species relative abundance by affecting plant functional traits in a Tibetan subalpine meadow
Source: Ecol Evol. 2021 Jul 27;11(16):11028–37. doi: 10.1002/ece3.7891 (PMC8366865; doi:10.1002/ece3.7891)
Supplement: Supplementary file 1 — Appendix S1 [file ECE3-11-11028-s001.doc]

**Text S1. Methods for aboveground and belowground trait measurements**

***Measurements of plant height, specific leaf area and photosynthetic rate***

According to the protocols and methods of Cornelissen et al. (2003), we measured plant height (the shortest distance between the upper boundary of the main photosynthetic tissue of the plant and the ground level) of 15 mature individuals for all species in each quadrat. Twenty of the second or third leaves from 15 mature individuals for all species in each quadrat were collected, stored in sealed plastic bags, and immediately transported to the laboratory. Leaf area (cm^2^) was determined using a scanner (CanoScan LiDE 700F), and analyzed with an image processing software (ImageJ, version 1.43u, National Institute of Mental Health, Bethesda, Maryland, USA). Leaves were then oven-dried at 80℃ for 2 days and weighed to determine leaf dry mass (W). Leaf specific area (SLA) was calculated as leaf area (cm^2^) per unit of W (g). At each site, the maximum photosynthetic rate of each species in each site was measured between 9:00 am and 12:00 am during sunny days, with a portable photosynthesis system (Li-6400, Li-Cor, Lincoln, Nebraska, USA). Based on preliminary trials, the photosynthetic photon flux density was set at 1500 µmol m^-2^ s^-1^ to ensure that light-saturated photosynthetic rates were measured for all species (Zhang et al., 2018). Ambient CO_2_ and air temperature were maintained at 370 µmol mol^-1^ and 26°C, respectively. 20 leaves from 15 mature individuals were selected per species for photosynthetic measurements.

***Measurements of below-ground trais (root density (RD), root biomass (RB), specific root length (SRL), specific root area (SRA) and root tissue density (RTD))***

15 mature individuals for all species found in both undisturbed and grazed meadows were carefully dug out with all above-ground and below-ground part having not any loss. Then below-ground parts for each individual for each species (Roots) were carefully washed by hand and root biomass was oven-dried at 80℃ for 48 h and weighed. The root traits length, surface area, volume and average diameter were measured using the WinRhizoTM scanner-based system (v.2007; Regent Instruments Inc., Quebec, Canada). For the analysis we used five root traits: root average diameter (RD; mm), root tissue density (RTD; root biomass per volume g/cm^-3^), specific root length (SRL; cm/g), and specific root surface area (SRA; cm^2^/mg ). We defined roots of the first and second order (diameter < 0.2 mm) as fine roots (McCormack et al., 2015) and calculated the percentage of fine roots (measured in length) as a proxy for absorptive capacity of the root system.

**References**

Cornelissen, J.H.C., Lavorel, S., Garnier, E., Diaz, S., Buchmann, N., Gurvich, D.E., Reich, P.B., ter Steege, H., Morgan, H.D., van der Heijden, M.G.A., Pausas, J.G., Poorter, H., 2003. A handbook of protocols for standardized and easy measurement of plant functional traits worldwide. Aust. J. Bot. 51, 335-380. https://doi.org/10.1071/bt02124

Zhang, H., John, R., Zhu, S.D., Liu, H., Xu, Q.Y., Qi, W., Liu, K., Chan, H., Ye, Q.,

2018. Shifts in functional trait–species abundance relationships over secondarysubalpine meadow succession in the Qinghai–Tibetan Plateau. Oecologia, 188, 41-47. https://doi.org/10.1007/s00442-018-4230-3

**Table S1.** The plant species collected in both intensively grazed meadow and undisturbed meadow with different life forms (annual (a) and perennial (p)). The tick and cross marks indicate species presence and absence, respectively.

| **Species name** | **Life form** | **Undisturbed meadow** | **Intensively grazed meadow** |
| --- | --- | --- | --- |
| *Aconitum carmichaeli* | a | *√* | *√* |
| *Agropyron cristatum* | p | *√* | *√* |
| *Agrostis gigantea* | p | *√* | *×* |
| *Agrostis trinii* | p | *√* | *×* |
| *Ajania tenuifolia* | a | *×* | *√* |
| *Ajuga lupulina* | p | *√* | *√* |
| *Anaphalis hancockii* | p | *√* | *√* |
| *Anaphalis lactea* | p | *√* | *×* |
| *Anemone obtusiloba* | p | *√* | *×* |
| *Anemone rivularis* | p | *√* | *√* |
| *Arenaria serpyllifolia* | p | *√* | *√* |
| *Artemisia annua* | a | *×* | *√* |
| *Artemisia desterorum* | p | *√* | *×* |
| *Artemisia tangutica* | p | *√* | *√* |
| *Aster alpinus* | p | *√* | *√* |
| *Astragalus polycladus* | p | *√* | *×* |
| *Bromus staintonii Meld* | p | *√* | *×* |
| *Carex kansuensis* | p | *√* | *√* |
| *Cerastium fontanum* | a | *√* | *√* |
| *Cirsium setosum* | p | *√* | *√* |
| *Delphinium kamaonense* | p | *√* | *×* |
| *Deschampsia caespitosa* | p | *√* | *×* |
| *Elymus nutans* | p | *√* | *×* |
| *Equisetum arvense* | p | *√* | *√* |
| *Euphorbia altotibetica* | p | *√* | *×* |
| *Equisetum ramosissimum* | a | *×* | *√* |
| *Euphorbia pekinensis* | a | *×* | *√* |
| *Euphrasia pectinata* | a | *×* | *√* |
| *Euphrasia regelii* | a | *√* | *√* |
| *Festuca rubra* | p | *√* | *×* |
| *Galium verum* | p | *√* | *×* |
| *Gentiana sino-ornata* | p | *√* | *×* |
| *Gentiana straminea* | p | *√* | *√* |
| *Gentianopsis paludosa* | a | *√* | *×* |
| *Geranium pylzowianum* | p | *√* | *×* |
| *Gueldenstaedtia multiflora* | p | *√* | *×* |
| *Halenia elliptica* | a | *×* | *√* |
| *Herminium monorchis* | a | *×* | *√* |
| *Heteropappus hispidus* | a | *√* | *√* |
| *Kobresia humilis* | p | *√* | *×* |
| *Koeleria cristata* | p | *√* | *×* |
| *Lancea tibetica* | p | *√* | *√* |
| *Leontopodium nanum* | p | *√* | *√* |
| *Leymus secalinus* | p | *√* | *×* |
| *Ligularia sagitta* | p | *√* | *√* |
| *Medicago ruthenica* | p | *√* | *√* |
| *Medicago sativa* | p | *√* | *×* |
| *Oxytropis kansuensis* | p | *√* | *×* |
| *Oxytropis ochrocephala* | p | *√* | *×* |
| *Pedicularis semitorta* | a | *√* | *√* |
| *Pedicularis semitorta* | a | *×* | *√* |
| *Plantago asiatica* | a | *√* | *√* |
| *Poa pachyantha* | p | *√* | *×* |
| *Poa pratensis* | p | *√* | *×* |
| *Polygonum sphaerostachyum* | a | *×* | *√* |
| *Polygonum viviparum* | a | *√* | *√* |
| *Potentilla anserina* | p | *√* | *√* |
| *Potentilla bifurca* | p | *√* | *√* |
| *Potentilla fragarioides* | p | *√* | *√* |
| *Potentilla potaninii* | p | *√* | *√* |
| *Roegneria kamoji* | p | *√* | *×* |
| *Roegneria nutans* | p | *√* | *×* |
| *Salvia roborowskii* | a | *×* | *√* |
| *Saussurea oblongifolia* | a | *×* | *√* |
| *Saussurea nigrescens* | p | *√* | *×* |
| *Saussurea parviflora* | p | *√* | *×* |
| *Scirpus pumilus* | p | *√* | *√* |
| *Sonchus transcaspicus* | a | *√* | *√* |
| *Stellera chamaejasme* | p | *√* | *√* |
| *Stipa aliena* | p | *√* | *×* |
| *Swertia tetraptera* | a | *√* | *√* |
| *Thermopsis lanceolata* | p | *√* | *×* |
| *Tibetia himalaica* | p | *√* | *×* |
| *Veronica eriogyne* | a | *×* | *√* |

**Table S2.** The long-transformed mean value of below-ground traits ((root average diameter (mm), root biomass (g), specific root

length (cm/g), specific root area (cm^2^/g) and root tissue density (g/cm^-3^)) and above-ground traits ((aboveground maximum

height (cm), leaf maximum photosynthesis rate (µmol m^-2^ s^-1^) and specific leaf area (cm^2^/g), and relative abundance (%) for

species with different life forms (annual (a) and perennial (p)) in the two different land use types (undisturbed meadow (U) and grazed meadow

(G)) respectively.

| **Species name** | **Life**  **form** | Relative  Abundance | Root  density | Root  biomass | Specific  root length | Specific  root area | Root tissue  density | Photosynthesis  rate | Aboveground  maximum height | Specific  leaf area | Land  use type |
| --- | --- | --- | --- | --- | --- | --- | --- | --- | --- | --- | --- |
| *Aconitum carmichaeli* | a | -4.382 | -0.400 | -2.996 | 4.998 | 4.395 | -3.912 | 2.203 | 1.609 | 5.678 | U |
| *Agropyron cristatum* | p | 0.759 | -1.966 | -1.050 | 2.168 | 2.629 | 0.239 | 2.393 | 4.105 | 5.007 | U |
| *Agrostis gigantea* | p | 0.419 | -2.120 | -0.916 | 1.852 | 2.500 | 0.419 | 1.788 | 3.068 | 5.239 | U |
| *Agrostis trinii* | p | 0.047 | -1.966 | 0.122 | 2.240 | 2.703 | 0.166 | 1.330 | 4.190 | 5.095 | U |
| *Ajuga lupulina* | p | -4.670 | -1.772 | -0.128 | 2.477 | 2.832 | -0.301 | 2.477 | 0.693 | 4.993 | U |
| *Anaphalis hancockii* | p | -1.779 | -0.211 | -3.507 | 5.108 | 4.492 | -4.605 | 2.349 | 3.332 | 5.014 | U |
| *Anaphalis lactea* | p | -6.056 | -0.821 | -2.120 | 4.191 | 4.017 | -2.813 | 2.061 | 2.140 | 5.632 | U |
| *Anemone obtusiloba* | p | -4.757 | -0.844 | -1.966 | 4.123 | 3.952 | -2.526 | 2.011 | 2.197 | 5.616 | U |
| *Anemone rivularis* | p | -2.376 | -1.204 | -1.238 | 3.353 | 3.429 | -1.833 | 1.889 | 2.603 | 5.427 | U |
| *Arenaria serpyllifolia* | p | 1.826 | -2.303 | -0.654 | 0.770 | -2.207 | 1.856 | 1.560 | 3.135 | 4.928 | U |
| *Artemisia desterorum* | p | 2.265 | -2.408 | -0.094 | 0.742 | 2.450 | 0.688 | 1.250 | 2.197 | 5.074 | U |
| *Artemisia tangutica* | p | -1.775 | -1.238 | -1.109 | 3.236 | 3.263 | -1.715 | 1.863 | 2.803 | 5.354 | U |
| *Aster alpinus* | p | -2.633 | -1.139 | -1.309 | 3.580 | 3.474 | -1.897 | 1.537 | 3.611 | 5.120 | U |
| *Astragalus polycladus* | p | -1.032 | -1.897 | -1.427 | 2.320 | 2.713 | 0.086 | 1.754 | 3.114 | 5.238 | U |
| *Bromus staintonii Meld* | p | 0.243 | -2.040 | -1.772 | 1.989 | 2.602 | 0.278 | 2.281 | 1.386 | 5.787 | U |
| *Carex kansuensis* | p | -0.053 | -2.207 | -0.073 | 1.250 | 1.522 | 1.384 | 1.984 | 2.303 | 5.576 | U |
| *Cerastium fontanum* | a | -4.447 | -0.261 | -3.219 | 5.011 | 4.468 | -4.605 | 2.203 | 1.609 | 5.699 | U |
| *Cirsium setosum* | p | -3.628 | -1.470 | -0.693 | 2.821 | 3.096 | -0.942 | 1.571 | 3.541 | 5.164 | U |
| *Delphinium kamaonense* | p | -1.283 | -1.386 | -0.942 | 3.021 | 3.163 | -1.273 | 2.217 | 1.609 | 5.701 | U |
| *Deschampsia caespitosa* | p | 0.521 | -2.040 | -0.386 | 1.947 | 2.591 | 0.336 | 1.506 | 4.159 | 4.902 | U |
| *Elymus nutans* | p | 0.688 | -2.120 | -2.408 | 1.708 | 2.375 | 0.432 | 2.365 | 1.792 | 5.014 | U |
| *Equisetum arvense* | p | -3.203 | -1.022 | -1.347 | 3.628 | 3.508 | -2.120 | 1.913 | 2.485 | 5.500 | U |
| *Euphorbia altotibetica* | p | -1.549 | -2.120 | -2.659 | 1.452 | 2.044 | 0.476 | 1.850 | 2.890 | 5.311 | U |
| *Euphrasia regelii* | a | -7.155 | 0.351 | -3.912 | 5.844 | 5.238 | -4.605 | 2.303 | 1.099 | 5.820 | U |
| *Festuca rubra* | p | 0.195 | -1.772 | -0.654 | 2.537 | 2.850 | -0.301 | 2.031 | 4.186 | 4.964 | U |
| *Galium verum* | p | -3.011 | -1.050 | -1.347 | 3.617 | 3.503 | -2.040 | 1.907 | 2.526 | 5.498 | U |
| *Gentiana sino-ornata* | p | -1.383 | -1.427 | -0.799 | 2.952 | 3.098 | -0.994 | 1.845 | 3.033 | 5.264 | U |
| *Gentiana straminea* | p | -3.571 | -0.994 | -1.470 | 3.691 | 3.592 | -2.120 | 1.966 | 2.351 | 5.560 | U |
| *Gentianopsis paludosa* | a | -2.376 | -0.545 | -2.659 | 4.993 | 4.383 | -3.507 | 2.202 | 1.792 | 5.673 | U |
| *Geranium pylzowianum* | p | -6.461 | -0.799 | -2.120 | 4.233 | 4.067 | -2.996 | 2.092 | 2.110 | 5.639 | U |
| *Gueldenstaedtia multiflora* | p | -1.100 | -1.273 | -1.022 | 3.114 | 3.194 | -1.427 | 1.353 | 2.079 | 5.119 | U |
| *Heteropappus hispidus* | a | -2.220 | -0.654 | -2.526 | 4.799 | 4.310 | -3.507 | 2.191 | 1.812 | 5.672 | U |
| *Kobresia humilis* | p | 2.913 | -2.659 | 1.247 | -2.996 | 2.236 | 2.104 | 1.051 | 4.277 | 4.901 | U |
| *Koeleria cristata* | p | 1.305 | -1.897 | -1.427 | 2.421 | 2.715 | 0.077 | 2.121 | 2.079 | 5.641 | U |
| *Lancea tibetica* | p | -3.203 | -0.994 | -1.427 | 3.663 | 3.560 | -2.120 | 1.954 | 2.398 | 5.533 | U |
| *Leontopodium nanum* | p | -4.064 | -0.868 | -1.897 | 4.001 | 3.897 | -2.303 | 1.988 | 2.303 | 5.602 | U |
| *Leymus secalinus* | p | 0.142 | -1.966 | -1.238 | 2.209 | 2.682 | 0.215 | 1.284 | 3.458 | 5.088 | U |
| *Ligularia sagitta* | p | -1.249 | -1.427 | -0.916 | 2.996 | 3.136 | -1.238 | 1.813 | 3.045 | 5.243 | U |
| *Medicago ruthenica* | p | -0.640 | -1.715 | -0.236 | 2.649 | 2.975 | -0.511 | 1.629 | 3.344 | 5.232 | U |
| *Medicago sativa* | p | -0.889 | -1.514 | -0.545 | 2.809 | 3.056 | -0.673 | 1.703 | 3.219 | 5.237 | U |
| *Oxytropis kansuensis* | p | -0.573 | -1.772 | -0.117 | 2.648 | 2.927 | -0.301 | 1.593 | 3.418 | 5.226 | U |
| *Oxytropis ochrocephala* | p | -0.985 | -1.470 | -0.693 | 2.817 | 3.077 | -0.916 | 2.006 | 2.282 | 5.614 | U |
| *Pedicularis semitorta* | a | -1.531 | -0.755 | -2.207 | 4.368 | 4.206 | -3.219 | 2.122 | 2.064 | 5.661 | U |
| *Plantago asiatica* | a | -2.410 | 0.095 | -3.912 | 5.647 | 4.938 | -3.912 | 1.890 | 2.590 | 5.452 | U |
| *Poa pachyantha* | p | 1.322 | -2.207 | 0.157 | 1.218 | 1.484 | 1.583 | 1.452 | 3.367 | 4.942 | U |
| *Poa pratensis* | p | -0.471 | -0.844 | -1.966 | 4.042 | 3.939 | -2.303 | 1.898 | 2.565 | 5.456 | U |
| *Polygonum viviparum* | a | -2.410 | -0.198 | -3.507 | 5.598 | 4.613 | -4.605 | 2.253 | 1.504 | 5.716 | U |
| *Potentilla anserina* | p | -1.380 | -1.347 | -0.994 | 3.078 | 3.188 | -1.273 | 1.845 | 3.033 | 5.275 | U |
| *Potentilla bifurca* | p | -2.633 | -1.109 | -1.309 | 3.610 | 3.499 | -1.966 | 1.901 | 2.565 | 5.460 | U |
| *Potentilla fragarioides* | p | -2.463 | -1.171 | -1.273 | 3.466 | 3.430 | -1.897 | 2.304 | 2.427 | 5.032 | U |
| *Potentilla potaninii* | p | -3.859 | -0.916 | -1.609 | 3.934 | 3.845 | -2.207 | 1.985 | 2.303 | 5.590 | U |
| *Roegneria kamoji* | p | -0.359 | -1.897 | -1.273 | 2.427 | 2.777 | 0.039 | 1.556 | 3.600 | 5.123 | U |
| *Roegneria nutans* | p | 1.319 | -2.120 | -0.654 | 1.697 | 2.358 | 0.457 | 2.338 | 1.386 | 5.028 | U |
| *Saussurea nigrescens* | p | -4.957 | -0.844 | -2.040 | 4.148 | 3.988 | -2.659 | 2.031 | 2.197 | 5.632 | U |
| *Saussurea parviflora* | p | -1.779 | -1.204 | -1.238 | 3.300 | 3.415 | -1.833 | 1.878 | 2.639 | 5.363 | U |
| *Scirpus pumilus* | p | -0.339 | -0.916 | -1.609 | 3.712 | 3.746 | -2.207 | 1.863 | 2.890 | 5.329 | U |
| *Sonchus transcaspicus* | a | -1.730 | -0.755 | -2.303 | 4.387 | 4.294 | -3.219 | 2.152 | 1.946 | 5.661 | U |
| *Stellera chamaejasme* | p | -6.461 | -1.273 | -1.079 | 3.148 | 3.218 | -1.514 | 1.870 | 2.773 | 5.359 | U |
| *Stipa aliena* | p | -0.431 | -1.833 | -0.062 | 2.465 | 2.797 | -0.073 | 1.571 | 3.570 | 5.126 | U |
| *Swertia tetraptera* | a | -2.392 | -0.799 | -2.207 | 4.258 | 4.158 | -2.996 | 1.583 | 3.448 | 5.199 | U |
| *Thermopsis lanceolata* | p | -0.841 | -1.561 | -0.261 | 2.740 | 3.008 | -0.654 | 1.688 | 3.248 | 5.233 | U |
| *Tibetia himalaica* | p | -0.571 | -1.238 | -1.139 | 3.275 | 3.368 | -1.715 | 1.744 | 3.114 | 5.237 | U |
| *Aconitum carmichaeli* | a | 1.853 | -0.288 | -4.605 | 6.844 | 6.637 | -3.219 | 4.057 | -0.494 | 6.655 | G |
| *Agropyron cristatum* | p | -4.852 | -3.912 | 4.928 | 5.161 | -2.659 | 3.963 | 2.579 | 2.653 | 5.884 | G |
| *Ajania tenuifolia* | a | -4.038 | -3.912 | 5.007 | 5.285 | -2.813 | 3.966 | 1.141 | 2.518 | 5.605 | G |
| *Ajuga lupulina* | p | -5.444 | -2.659 | 3.979 | 4.518 | -0.868 | 3.476 | 2.287 | 3.540 | 5.744 | G |
| *Anaphalis hancockii* | p | -5.432 | -2.659 | -0.942 | 2.074 | 3.374 | -0.186 | 3.189 | 3.599 | 5.583 | G |
| *Anemone rivularis* | p | -0.074 | -2.408 | -4.605 | 5.311 | 5.943 | -3.912 | 3.727 | 1.679 | 6.246 | G |
| *Arenaria serpyllifolia* | a | -3.234 | -4.605 | 5.708 | 3.957 | -3.507 | 4.084 | 0.867 | 3.028 | 5.780 | G |
| *Artemisia annua* | a | 1.781 | -1.514 | -2.408 | 6.764 | 4.305 | -0.734 | 3.897 | 0.802 | 6.600 | G |
| *Artemisia tangutica* | p | -4.885 | -2.996 | -2.996 | 4.277 | 4.829 | -1.661 | 3.427 | 2.802 | 5.936 | G |
| *Aster alpinus* | p | -1.285 | -2.526 | -2.996 | 5.141 | 4.806 | -1.470 | 3.852 | 1.696 | 6.210 | G |
| *Carex kansuensis* | p | -5.288 | -2.408 | 6.073 | 4.188 | -4.605 | 4.355 | 2.362 | 2.971 | 5.817 | G |
| *Cerastium fontanum* | a | -3.839 | -3.912 | 5.088 | 3.009 | -2.996 | 4.027 | 1.141 | 2.376 | 5.731 | G |
| *Cnidium monnieri* | a | 1.528 | -1.715 | -3.912 | 6.016 | 5.300 | -2.996 | 3.795 | 1.197 | 6.482 | G |
| *Equisetum arvense* | p | -5.108 | -3.507 | -2.040 | 4.679 | 4.172 | -0.511 | 3.704 | 2.856 | 5.909 | G |
| *Equisetum ramosissimum* | a | 1.235 | -1.833 | -3.507 | 5.935 | 5.078 | -2.408 | 3.768 | 1.364 | 6.450 | G |
| *Euphorbia pekinensis* | a | -2.999 | -4.605 | 5.897 | 4.010 | -3.507 | 4.101 | 2.958 | 3.135 | 5.803 | G |
| *Euphrasia pectinata* | a | -4.135 | -3.912 | 4.906 | 5.130 | -2.659 | 3.932 | 2.590 | 2.601 | 5.696 | G |
| *Euphrasia regelii* | a | -3.599 | -4.605 | 5.237 | 4.417 | -3.219 | 4.057 | 1.089 | 2.687 | 5.748 | G |
| *Gentiana straminea* | p | -4.719 | -2.813 | -1.514 | 3.133 | 3.965 | -0.274 | 3.466 | 3.133 | 5.968 | G |
| *Halenia elliptica* | a | 0.933 | -2.120 | -3.219 | 5.461 | 4.937 | -2.040 | 3.767 | 1.579 | 6.326 | G |
| *Herminium monorchis* | a | -3.524 | -4.605 | 5.264 | 2.618 | -3.219 | 4.069 | 2.558 | 3.262 | 5.754 | G |
| *Heteropappus hispidus* | a | 1.624 | -1.661 | -2.996 | 6.106 | 4.825 | -1.514 | 3.805 | 1.109 | 6.584 | G |
| *Lancea tibetica* | p | -6.461 | -3.507 | 2.417 | 3.669 | 0.329 | 2.957 | 2.992 | 4.019 | 5.181 | G |
| *Leontopodium nanum* | p | -2.298 | -2.996 | -2.813 | 3.718 | 4.715 | -1.273 | 3.535 | 1.783 | 6.134 | G |
| *Ligularia sagitta* | p | -2.939 | -3.219 | 6.028 | 4.026 | -4.605 | 4.272 | 3.437 | 2.965 | 5.827 | G |
| *Medicago ruthenica* | p | -6.442 | -2.303 | -0.755 | 1.957 | 2.677 | -0.062 | 3.189 | 3.793 | 5.581 | G |
| *Pedicularis kansuensis* | a | -4.459 | -3.219 | 4.192 | 4.586 | -1.238 | 3.659 | 2.844 | 3.581 | 5.782 | G |
| *Pedicularis semitorta* | a | -4.541 | -2.303 | -2.659 | 4.445 | 4.566 | -1.109 | 3.171 | 2.134 | 6.033 | G |
| *Plantago asiatica* | a | -4.466 | -2.996 | -2.659 | 4.382 | 4.575 | -1.171 | 3.470 | 2.054 | 6.048 | G |
| *Polygonum sphaerostachyum* | a | -4.210 | -3.912 | 4.902 | 5.119 | -2.526 | 3.910 | 1.322 | 3.410 | 5.667 | G |
| *Polygonum viviparum* | a | -4.550 | -2.813 | -2.526 | 4.318 | 4.563 | -1.050 | 3.235 | 2.224 | 5.992 | G |
| *Potentilla anserina* | p | -2.672 | -2.659 | -3.219 | 4.568 | 5.025 | -2.303 | 3.513 | 1.369 | 6.088 | G |
| *Potentilla bifurca* | p | -5.538 | -3.507 | 3.694 | 4.275 | -0.635 | 3.461 | 2.347 | 3.602 | 5.681 | G |
| *Potentilla fragarioides* | p | -2.155 | -2.526 | -2.120 | 4.791 | 4.259 | -0.528 | 3.540 | 1.735 | 6.177 | G |
| *Potentilla potaninii* | p | -0.785 | -2.526 | -2.813 | 5.299 | 4.734 | -1.427 | 3.694 | 1.696 | 6.236 | G |
| *Salvia roborowskii* | a | -4.564 | -2.813 | -2.408 | 4.376 | 4.386 | -0.821 | 3.769 | 2.318 | 6.004 | G |
| *Saussurea oblongifolia* | a | -4.318 | -3.507 | 4.582 | 5.020 | -2.207 | 3.812 | 1.445 | 3.480 | 5.937 | G |
| *Scirpus pumilus* | p | -5.800 | -2.408 | 2.925 | 4.006 | -0.386 | 3.356 | 2.635 | 3.659 | 5.663 | G |
| *Sonchus transcaspicus* | a | -4.308 | -3.507 | 4.755 | 5.074 | -2.408 | 3.888 | 2.294 | 3.471 | 5.675 | G |
| *Stellera chamaejasme* | p | 0.552 | -2.303 | -2.996 | 5.359 | 4.884 | -1.715 | 3.750 | 1.633 | 6.297 | G |
| *Swertia tetraptera* | a | -4.332 | -3.507 | 4.545 | 4.892 | -1.833 | 3.805 | 2.502 | 3.462 | 5.919 | G |
| *Veronica eriogyne* | a | -4.353 | -3.219 | -2.659 | 4.198 | 4.685 | -1.273 | 3.686 | 3.475 | 5.786 | G |

**Table S3.** Principal component analysis (PCA) for the first two principal components

for all species based on 8 plant traits (root average diameter (mm), root biomass (g), specific root length (cm/g), specific root area (cm^2^/g), root tissue density (g/mm^3^), maximum photosynthesis rate (µmol m^-2^ s^-1^), height (m), and specific leaf area (cm^2^/g)) of all species. PC loadings and the percentage of variance explained by the first two PCs are reported.

| **Traits** | **PC1** | **PC2** |
| --- | --- | --- |
| Root average diameter | -0.35 | 0.37 |
| Root biomass | 0.45 | -0.19 |
| Specific root length | -0.22 | -0.43 |
| Specific root area | -0.45 | 0.18 |
| Root tissue density | 0.45 | -0.22 |
| Photosynthesis rate | -0.24 | -0.44 |
| Abovrground maximum height | 0.35 | 0.23 |
| Specific leaf area | -0.20 | -0.56 |

**Fig. S1.** The topographic map of our study site in the eastern part of the Qinghai-Tibetan Plateau, China (A), and the quadrat sampling design (B).


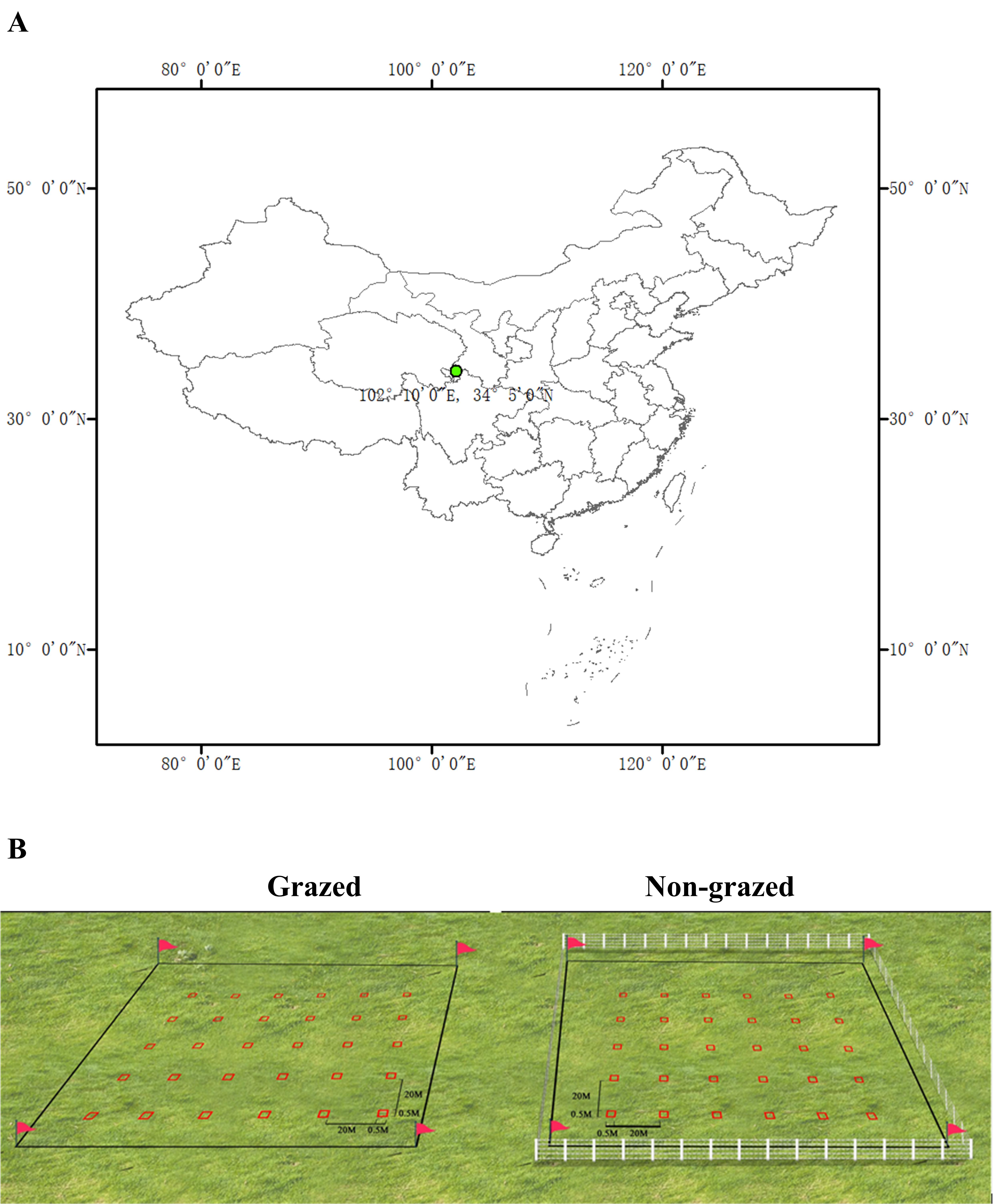


**Fig. S2.** Differences in aboveground biomass (g) between annual and perennial species in non-grazed and grazed meadows respectively. *** indicates P<0.001 based on Wilcoxon signed-rank tests.


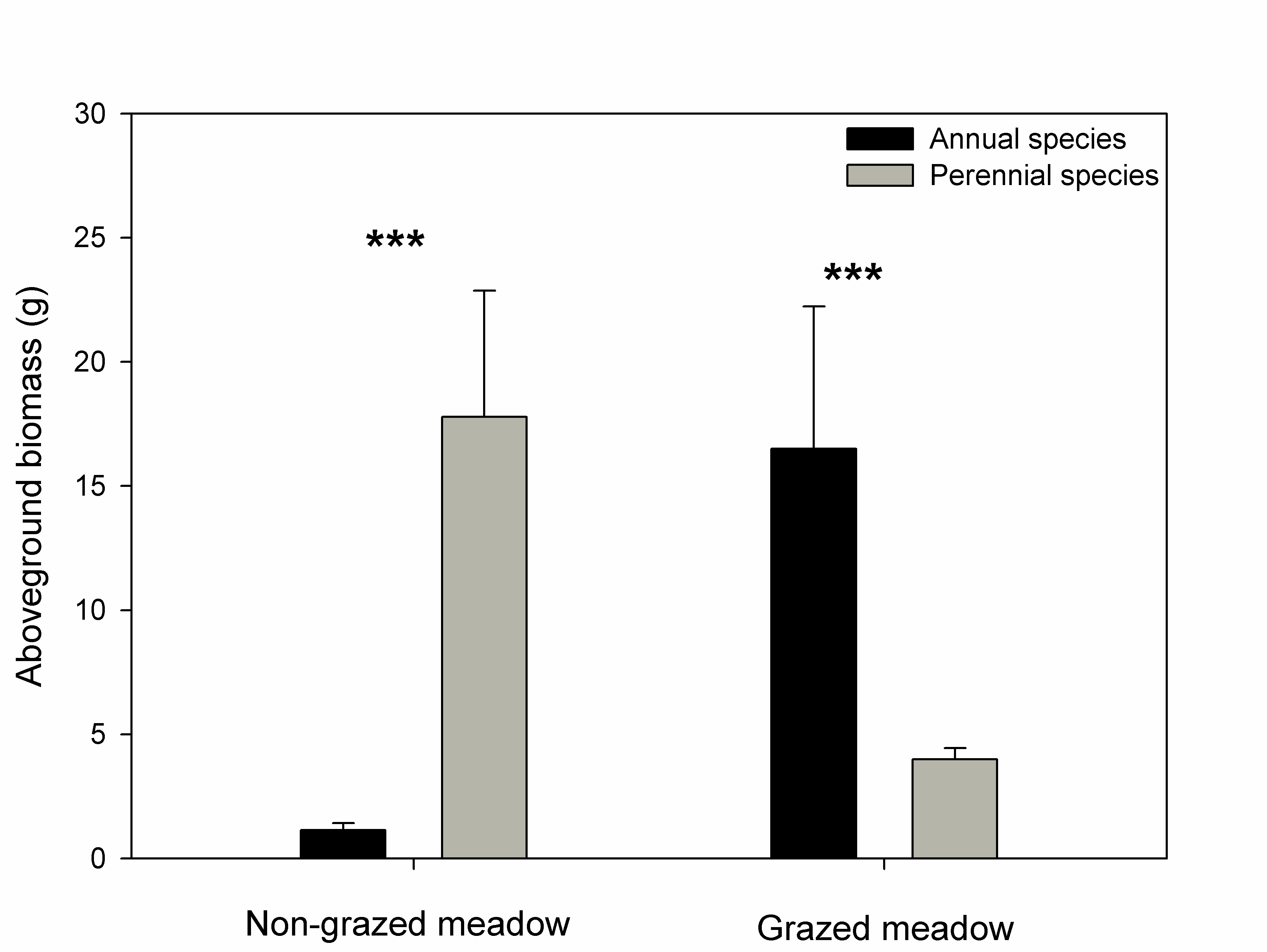


**Fig. S3.** Relationships between principal component analysis (PCA) axis 2 (PC2) and species relative abundance in non-grazed and grazed meadows. Fitted lines are generated from linear regression with corresponding significance (P).
